# A Preliminary Evaluation of Transdiagnostic Group Metacognitive Therapy in a Mixed Psychological Disorder Sample

**DOI:** 10.3389/fpsyg.2019.01341

**Published:** 2019-06-20

**Authors:** Pia Callesen, Lora Capobianco, Calvin Heal, Carsten Juul, Sisse Find Nielsen, Adrian Wells

**Affiliations:** ^1^School of Health Sciences, Division of Psychology and Mental Health, University of Manchester, Manchester, United Kingdom; ^2^CEKTOS, Copenhagen, Denmark; ^3^Research and Innovation, Greater Manchester Mental Health NHS Foundation Trust, Manchester, United Kingdom; ^4^School of Health Sciences, Division of Population Health, Health Services Research and Primary Care, Manchester, United Kingdom

**Keywords:** metacognitive therapy, transdiagnostic, depression, anxiety, group therapy

## Abstract

**Objective:** Comorbidity is common among anxiety and depression. Transdiagnostic treatment approaches have been developed to optimize treatment and offer a more unified approach suitable for individuals with comorbidities. Metacognitive therapy (MCT) is a transdiagnostic therapy for psychological disorder and is based on the metacognitive model. The present study is a service evaluation of the outcomes associated with group MCT delivered to unselected patients at a Danish outpatient clinic.

**Methods:** A total of 131 self-diagnosed patients received 6 sessions of group MCT. Symptoms of anxiety and depression were measured by the Hospital Anxiety and Depression scale (HADS) and metacognition was assessed using the Cognitive Attentional Syndrome-1 (CAS-1). Participants were assessed at pre-treatment, post-treatment, and at 6 months follow-up as per usual clinic protocol. Linear mixed-effects regressions were used to assess the transdiagnostic effects of group MCT. Treatment effect sizes are reported for subgroups based on participant’s reason for seeking treatment (anxiety, depression, or comorbid). Effect sizes were not conducted for the depression subgroup given the limited number of participants. Clinically significant change is reported for all subgroups.

**Results:** Group MCT was associated with large effect sizes for symptoms of anxiety and depression for patients seeking treatment for anxiety (*d* = 1.68), or comorbid (1.82). In addition, 66.7% of patients were classified as recovered at post-treatment, and 12.9% were classified as improved. These results were largely maintained at 6-month follow-up.

**Conclusion:** These preliminary findings support the continued use of group MCT in the current outpatient clinic and suggest that it may be an efficacious and cost-effective treatment when delivered in “transdiagnostic” groups.

## Introduction

There is increasing evidence demonstrating that anxiety and depression rarely occur alone and instead are highly comorbid (Brown et al., 2001). Brown et al. (2001) evaluated the current and lifetime comorbidity of anxiety and mood disorders and highlighted that, of those with a principal anxiety or mood disorder, the current and lifetime comorbidity with other Axis I disorders was 57 and 81% respectively. Similarly, Lamers et al. (2011) investigated the comorbidity patterns of anxiety and depression in the Netherlands and found that 67% of patients with a depressive disorder had a current comorbid anxiety disorder. Furthermore, among individuals with a current anxiety disorder, 63% had current comorbid depressive disorder. Despite the high rate of comorbidity, psychological paradigms such as Cognitive Behavioral Therapy (CBT) often focus on providing a disorder-specific treatment, whereby separate protocols are used for treating different disorders such as generalized anxiety, OCD, PTSD, and depression. These protocols are typically supported by disorder-specific case formulations and models. However, treatments focusing on disorder-specific models can be problematic as patients often do not present with a single disorder. Therefore, clinicians are required to treat the most pressing disorder even though the patient may be presenting with more than one problem.

The high comorbidity rate among mental disorders supports the need for transdiagnostic models and treatments that focus on the common underlying processes that maintain psychological disorders. CBT is one of the most widely evaluated treatments for psychological disorders. Although CBT is primarily delivered using a disorder-specific protocol, more recent research has aimed to deliver CBT using a transdiagnostic approach. A recent systematic review and meta-analysis of transdiagnostic CBT for anxiety and depression found mixed results on its effectiveness. Of the two studies that compared transdiagnostic CBT to a control condition, only one study (Schmidt et al., 2012) found some evidence for the effectiveness of this approach, while Erickson et al. (2007) did not report significant findings for anxiety.

One transdiagnostic approach to CBT is the unified protocol (UP) for emotional disorders (Barlow et al., 2010). The UP incorporates principles from traditional CBT such as cognitive restructuring and exposure procedures together with advances in emotion regulation research, including an emphasis on increasing patient’s awareness of maladaptive cognitions and behaviors (Wilamowska et al., 2010; Craske, 2012; Farchione et al., 2012; Bullis et al., 2015; Laposa et al., 2017). Bullis et al. (2015) evaluated the UP in a group format delivered over 12 sessions. The authors demonstrated medium to large effect sizes on symptom measures of depression and anxiety respectively. However, the study had a small sample size of 11 participants. More recently, Laposa et al. (2017) evaluated the UP in a group format over 14 sessions with 26 participants. There were medium to large effect sizes on measures of anxiety and depression but they noted that participant’s scores on the Penn State Worry Questionnaire and Social Interaction Anxiety Scale remained above their clinical cutoffs at post-treatment. Furthermore, the Depression Anxiety Stress Scale-Anxiety sub scale and Quick Inventory of Depressive Symptom scores remained in the moderate range at the end of treatment.

In other areas, third-wave approaches of behavioral and cognitive behavioral therapies such as mindfulness-based stress reduction (MBSR; Kabat-Zinn, 1990) and acceptance and commitment therapy (ACT; Hayes et al., 1999) have also been used to treat transdiagnostic samples. MBSR focuses on cultivating present moment awareness and combines formal and informal mindfulness practices such as mindfulness of the breath, thoughts, bodily sensations, and routine activities. ACT combines psychoeducation with exercises that aim to increase mental flexibility and mindfulness experiences while decreasing avoidance of activities. ACT targets six core processes with the aim of increasing psychological flexibility. The six core processes are: contact with the present moment, values, committed action, self as context, delusion, and acceptance. ACT integrates mindfulness and acceptance processes and commitment and behavior change processes to enhance psychological flexibility (Hayes et al., 2006). In a recent systematic review and meta-analysis, Newby et al. (2015) found a significant difference favoring CBT in comparison to mindfulness/acceptance-based interventions in anxiety symptoms. (CBT, *hedge’s g* = 0.88, mindfulness/acceptance, hedge’s *g* = 0.61). However, there was no significant difference between treatment type on symptoms of depression (CBT, hedge’s *g* = 0.84, mindfulness/acceptance, hedge’s *g* = 0.92).

One of the earlier transdiagnostic approaches was presented by Wells and Matthews (1994, 1996) in their Self-Regulatory Executive Function (S-REF) model. They argued for the conceptualization of universal psychological factors across pathologies and asserted that psychological disorder is maintained by a common maladaptive cognitive attentional syndrome (CAS) that should be the target of treatment. The CAS is characterized by increased self-focused attention, repetitive negative thinking involving worry and rumination, and unhelpful coping strategies and behaviors such as attentional threat monitoring, thought suppression, and avoidance. The CAS is a result of an individual’s metacognitive beliefs which lead to prolonged negative processing and consequent distress. There are two types of metacognitive beliefs: positive metacognitive beliefs (PMC) and negative metacognitive beliefs (NMC). Negative metacognitive beliefs concern the uncontrollability and danger of worry (i.e., “I cannot control my worry,” “my worrying may harm me”). In contrast, positive metacognitive beliefs concern the usefulness of worry (i.e., “worrying helps me cope,” “if I worry I’ll be prepared). These underlying metacognitive beliefs are considered a major factor driving the CAS. Metacognitive therapy (MCT: Wells, 1995, 2009) was developed based on this model, and aims to remove the CAS and modify positive and negative metacognitive beliefs. MCT has demonstrated significant efficacy across various psychological disorders. Normann et al. (2014) conducted a meta-analysis evaluating the efficacy of MCT for anxiety and depression, where they reported that MCT was a highly effective treatment. When MCT was compared to wait list control on the primary outcome measure, effect sizes favored MCT, *g* = 1.81. In addition, when MCT was compared with CBT, a large effect size was found favoring MCT, *g* = 0.97. Recently, Normann and Morina (2018) conducted an updated systematic review and meta-analysis on MCT for anxiety and depression and found that, when MCT was compared to wait list controls, there was a large pre- to post-treatment effect size, *g* = 2.06. Similarly, when MCT was compared to active control treatments, there was a medium to large effect size in favor of MCT, *g* = 0.68. More specifically, when MCT was compared to cognitive behavior therapy and behavioral activation interventions, a medium to large effect size was found favoring MCT from pre- to post-treatment, *g* = 0.69. Although MCT has been evaluated using an individual treatment format, there is increasing evidence that MCT is effective using a group format. McEvoy et al. (2015) tested group MCT in individuals with GAD who received six sessions of treatment for 2 h plus an additional 1-month follow-up session. The authors found that group MCT was associated with very large effect sizes from pre- to post-treatment on measures of negative metacognitions, worry, and repetitive negative thinking (*d* = 1.75–1.90). In addition, when evaluating reliable and clinically significant change based on Jacobson and Truax (1991) criteria, they found that at post-treatment, 86% of patients had reliably improved and 74% had recovered. Preliminary studies also highlight the efficacy of MCT in transdiagnostic samples (Johnson and Hoffart, 2016; Hagen et al., 2017; Johnson et al., 2017; Capobianco et al., 2018). Johnson et al. (2017) compared transdiagnostic MCT in an individual format with disorder-specific CBT and found that MCT was more effective than CBT (Cohen’s *d* = 0.7) in alleviating anxiety symptoms at post-treatment. There was no difference at 12-month follow-up but this may be due to patients accessing other treatments over this period. Capobianco et al. (2018) conducted a pilot feasibility study comparing group-delivered MCT or MBSR. They noted that while both treatments were acceptable and feasible to deliver in a group format, the preliminary data suggested that MCT might be more effective than MBSR.

In the present study, we aimed to add to the data on the effects associated with transdiagnostic MCT by collating the outcome data of patients who entered into group therapy in a Danish primary care outpatient clinic. The data that were routinely collected allowed us to examine the effects associated with receiving group MCT in a group of individuals self-reporting their reason for seeking treatment in a standard outpatient care setting. Such liberal inclusion criteria and the setting of the treatment are especially informative because they overcome one of the criticisms of tightly controlled trials that use extensive inclusion/exclusion criteria, thus compromising the extent to which participants represent those who are typically seen in outpatient clinics.

## Materials and Methods

### Design

The design is essentially a service audit and is therefore an uncontrolled pre-post assessment with 6 months follow-up. Participants attended the Center for Cognitive Therapy and Supervision (CEKTOS), a Danish primary care outpatient clinic. Ethical approval was not sought for the study as data were collected as part of routine clinical practice and evaluated as part of a service audit. However, in accordance with clinical guidelines, patients provided informed and written consent for use of patient data and ethical standards for reporting were adhered to. This is in line with the rules and regulations of the Danish National Ethics Committee. As new patients contacted the clinic, they were offered the choice of group therapy or individual therapy. Recruitment occurred between August 2014 and May 2015; during this time, a total of 145 patients opted to take part in the group therapy being offered, which was 21% uptake rate for group therapy. The Generalized Anxiety Disorder Assessment (GAD-7) and Patient Health Questionnaire (PHQ-9) were administered at pre-treatment as part of general assessment and are reported here to help describe the sample. The Hospital Anxiety and Depression Scale (HADS) and CAS-1 were administered at pre-treatment, mid-treatment, post-treatment, and 6 months follow-up. Participants who opted to take part in group therapy were later approached and asked to provide written and informed consent to allow their anonymized data to be released for use in this evaluation; 14 participants (10%) did not consent for their data to be used for analyses. In addition, seven participants were removed from the analysis as they did not report a reason for seeking treatment, resulting in a total sample of 124 participants. Participants were given the opportunity to withdraw from the treatment at any time during the treatment and follow-up.

### Participants

There were 124 Danish outpatients (87 women, 37 men) treated. The mean age of the total sample was 42.10 (SD = 12.73; age range: 18–68). A total of 51 participants were currently taking medication for anxiety or depression. As there was less than 5% of missing data, means were used for imputing missing values. There was no intake interview or screening of suitability, this was an open treatment in which all consenting patients were deemed suitable and both referred and non-referred clients were eligible. Patients represented a range of different disorders.

### Procedure

Participants completed 6 weeks of group metacognitive therapy. Sessions lasted approximately 2 h. There were 16 groups with an average of eight participants in each. The outcome measures were administered at pre-, mid- and post-treatment, with 3 weeks between questionnaire administrations.

## Measures

### Primary Outcome Measure

#### Hospital Anxiety and Depression Scale

The *Hospital Anxiety and Depression Scale* (HADS; Zigmond and Snaith, 1983) is a 14-item scale with two subscales (anxiety and depression). Each item is scored from 0 to 3, with subscale scores greater than 8 being indicative of anxiety and depression. Both subscales demonstrate good internal consistency, good validity and reliability (Zigmond and Snaith, 1983; Herrmann, 1997; Mykletun et al., 2001).

### Secondary Outcome Measure

#### Cognitive Attentional Syndrome

The *Cognitive Attentional Syndrome* (CAS-1; Wells, 2009) assesses the extent to which the cognitive attentional syndrome, a key component of the metacognitive model, is activated in the last week. The CAS-1 is a 16-item measure where the first eight items are rated on a scale from 0 to 8, where 0 indicates none of the time and 8 indicates all of the time. Items 1 and 2 assess the extent to which individuals have been dwelling, worrying, or focusing on possible threat in the past week. Items 3–8 assess various coping behaviors that individuals may be engaging in to deal with negative thoughts (e.g., tried to control emotions, asked for reassurance). The final item assesses the positive and negative metacognitive beliefs that individuals hold (e.g., “*I cannot control my thoughts*,” “*analysing my problems will help me find answers*”). The CAS-I demonstrates good internal consistency (Cronbach’s alpha = 0.86) (Fergus et al., 2012).

### Pre-treatment Screening Measures

#### Patient Health Questionnaire

This is a 9-item measure that assesses depression in primary health care, where greater scores indicate increasing severity of symptoms (Kroenke et al., 2001). Items are rated on a scale from 0 (not at all) to 3 (nearly every day). The scale has four cutoff points: 5 (mild depression), 10 (moderate depression), 15 (moderately severe depression), and 20 (severe depression). The scale demonstrates good reliability and validity (Cameron et al., 2008).

#### Generalized Anxiety Disorder Assessment

This is a brief 7-item measure used to assess symptoms of generalized anxiety disorder in primary health care (Spitzer et al., 2006). Items are rated on a scale from 0 (not at all) to 3 (nearly every day), with greater scores indicating greater severity of anxiety. The scale has three cutoff points: 5 (mild anxiety), 10 (moderate anxiety), and 15 (severe anxiety). The scale demonstrates good internal and test-retest reliability, and good convergent and construct validity (Spitzer et al., 2006).

### Intervention

Group MCT was supported by the Generalized Anxiety Disorder (GAD) protocol as described in the treatment manual by Wells (2009) as this represents the core of the transdiagnostic treatment. The attention training technique (ATT, Wells, 1990) was added in the group treatment sessions, as the ATT helps to address perseverative thinking by promoting attention flexibility and executive control skills and is often used in depression. The ATT also meets the 5-3-20 criterion (Kratochwill et al., 2013) for an evidence-based intervention (Rochat et al., 2018). The 5-3-20 criterion states that an intervention is evidence based if it meets the following criteria: (1) the intervention has a minimum of five single case design studies that either meets standards or meets standards with reservations; (2) The single case design studies are conducted by at least three research teams with no overlapping authorship at three different institutions; and (3) the total number of cases (i.e., participants, classrooms, etc.) across studies totals at least 20. Sessions were delivered by two clinical psychologists trained in MCT and who were supervised by AW. Participants received six weekly sessions of group MCT that lasted approximately 2 h. Sessions focused on a group case formulation, the attention training technique, detached mindfulness, challenging positive and negative metacognitive beliefs, and formulating a personalized plan B. The plan B allowed participants to consolidate what they had learned in therapy and have a summary of how to deal with future negative cognitions.

### Statistical Analysis Plan

Analyses were conducted in STATA (version 15). Multiple imputation was used to impute missing data. Categorical variables were assessed using a Chi-square test. A linear mixed-effects regression incorporating all three time points (pre-treatment, post-treatment, and follow-up) on the total sample (ITT) was applied in order to evaluate the significance of change overall and examine any modifying effects of type of problem on outcome. A sensitivity analysis was also conducted; we used mean imputation to impute missing values at follow-up where missingness was 25.6% for each of the three outcomes. There was less than 1% missingness at pre- and post–treatment, so no imputation was used for these time points. Finally, effect sizes from pre-treatment to post-treatment and pre-treatment to follow-up were based on completers and calculated as Cohen’s *d* (Cohen, 1988) using the formula *d* = (*M*_1_−*M*_2_)/SD_pooled_, where *M*_1_ is the mean at pre-treatment, *M*_2_ is the mean at post-treatment or follow-up, and SD_pooled_ is the pooled standard deviation. We used the method outlined by Jacobson and Truax (1991) to calculate reliable clinical change based on the HADS total score, with the cut-off score being calculated using criterion “c,” which was only conducted on treatment completers. Individuals were classified as recovered if they made a reliable change and were below the cut-off score. Individuals were classified as improved if they made a reliable change but were not below the cut-off score a post-treatment or follow-up. As the sample size for depression subgroup from pre- to post-treatment (*n* = 12) and pre-treatment to follow-up (*n* = 8) was disproportionately smaller than that of the other subgroups, it was not included in the effect size calculation.

## Results

**Figure 1:**
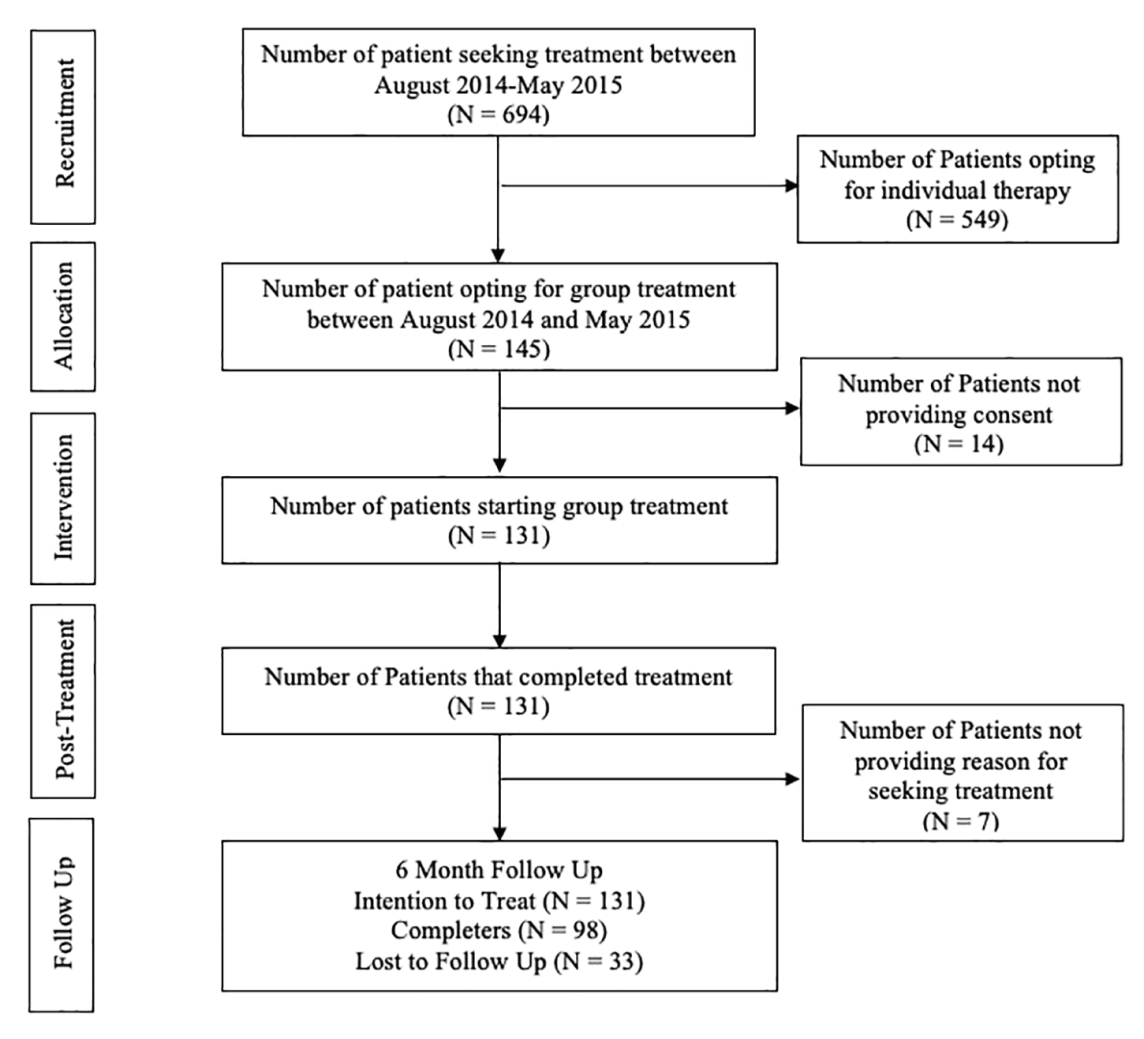
CONSORT diagram.

The flowchart (Figure 1) shows number of patients contacting the clinic for help, and the number of patients entering the transdiagnostic group intervention. Approximately 9–14 patients call the clinic each day, and of those, approximately 21% chose to participate in the group, while 79% chose to complete individual therapy. Completer analysis was conducted at post-treatment. At follow-up, there was a 70% data return rate.

### Descriptive Statistics

Table 1 highlights the characteristics of participants based on their self-reported reason for seeking treatment. The total sample included 124 patients, 37 males (29.8%) and 87 females (70.2%). Individuals reported their primary reason for seeking treatment as anxiety, depression, or both (Table 1). Participants also reported secondary reasons for seeking treatment which included stress (58 participants), obsessive compulsive disorder (OCD; nine participants), and post-traumatic stress disorder (PTSD; one participant); however, six participants reported secondary reasons for seeking treatment as stress and obsessive compulsive disorder, and an additional six participants reported secondary reasons for seeking treatment as stress and PTSD. All subsequent analyses are based on individual’s primary reason for seeking treatment. A Chi-square analysis demonstrated that there was a significant difference in primary reason for seeking treatment (e.g., anxiety, depression, both) by gender, *χ*^2^ (2, *N* = 124) = 9.76, *p* = 0.008. There was a greater number of females seeking treatment for anxiety in comparison to males [52 females (41.9%), 11 males (8.9%)], with a similar pattern for those seeking treatment for anxiety and depression [29 females (23.4%), 20 males (16.1%)], while there was an equal gender balance for those seeking treatment for depression (9.7%). Ninety-three participants completed questionnaires at 6-month follow-up [66 women (71.0%), 27 men (29.0%)]. Table 2 provides an overview of the means and standard deviations for the outcome measures at pre-treatment, post-treatment, and follow-up based on individuals’ self-reported diagnosis and for the total sample of treatment completers.

**Table 1:** Descriptive statistics by reason for seeking treatment.

	Anxiety (*N* = 63)	Depression (*N* = 12)	Comorbid (*N* = 49)	Total sample (*N* = 124)
Gender (M:F)	11:52	6:6	20:29	37:87
Age (M(SD))	41. 14 (12.48)	36.75 (14.47)	44.63 (12.29)	42.10 (12.73)
PHQ-9 (M(SD))	11.37 (5.59)	15.42 (4.83)	14.33 (4.34)	12.93 (5.28)
GAD-7 (M(SD))	12.28 (5.46)	11.83 (3.16)	12.90 (4.50)	12.47 (4.89)

Note: M = mean; SD = standard deviation.

**Table 2:** Means and standard deviations for outcome measures for all patients (ITT) and treatment completers by reason for seeking treatment.

	Pre-treatment (ITT)	Post-treatment (ITT)	Follow up (COMPLETERS)
**HADS-anxiety**			
Anxiety Depression Comorbid Total sample	12.89 (3.97) 11.67 (3.47) 13.12 (3.31) 12.86 (3.67)	7.14 (3.25) 6.17 (3.10) 7.14 (2.96) 7.05 (3.11)	7.04 (5.13) 4.25 (3.53) 6.47 (3.50) 6.79 (4.46)
**HADS-depression**			
Anxiety Depression Comorbid Total sample	7.84 (3.84) 10.67 (2.57) 10.53 (3.93) 9.18 (3.99)	3.54 (3.05) 4.50 (3.34) 4.82 (3.87) 4.14 (3.45)	3.83 (4.29) 2.63 (3.66) 4.82 (4.05) 4.27 (4.19)
**HADS-total**			
Anxiety Depression Comorbid Total sample	20.73 (6.79) 22.33 (2.99) 23.65 (6.39) 22.40 (6.47)	10.68 (5.69) 10.67 (5.74) 11.96 (6.08) 11.19 (5.84)	10.87 (8.86) 6.88 (6.98) 11.29 (6.93) 11.06 (8.01)
**PMC**			
Anxiety Depression Comorbid Total sample	193.33 (69.78) 189.00 (74.87) 178.53 (68.0) 187.06 (69.35)	51.41 (59.45) 44.67 (70.02) 52.65 (61.40) 51.25 (60.79)	57.87 (68.75) 61.25 (103.43) 50.08 (57.62) 54.39 (63.77)
**NMC**			
Anxiety Depression Comorbid Total sample	193.90 (80.00) 211.67 (63.33) 218.47 (75.59) 205.33 (77.16)	40.13 (44.99) 99.58 (91.66) 64.35 (81.85) 55.45 (68.76)	61.06 (80.02) 80.63 (71.34) 70.66 (81.35) 65.35 (80.28)

Note: HADS = hospital anxiety and depression scale; CAS-1 = cognitive attentional syndrome 1; PMC = positive metacognitions; NMC = negative metacognitions; M = mean; SD = standard deviation.

### Outcomes Associated With Group Treatment

#### Hospital Anxiety and Depression Scale

To assess if there were any differences between groups (anxiety, depression, comorbid) over time, a mixed-effect regression was conducted. There was a significant main effect of time, with post-treatment and 6-month follow-up being associated with a respective 10.9 (95% CI 9.7–12.0) and 11.2 (95% CI 9.5–12.9) point reduction in HADS total score compared to baseline (*p* < 0.001), for the entire sample. There were nonsignificant differences between groups on HADS total score at post-treatment, for the depression compared to anxiety group [−1.62 (95% CI −4.68 to 1.44), *p* = 0.300], for the comorbid group compared to anxiety group [−1.65 (95% CI −4.21 to 0.91), *p* = 0.208], and for the comorbid group compared to depression group [−0.03 (95% CI −3.31 to 3.25), *p* = 0.987]. At follow-up however, there was a significant difference between the depression and anxiety groups [−6.48 (95% CI −11.43 to −1.53), *p* = 0.010]. This suggests that the depression subgroup had improved significantly more than the anxiety subgroup by follow-up. There was no such significant difference between the comorbid group and anxiety group [−3.08 (95% CI −6.64 to 0.47), *p* = 0.089] at follow-up, nor between the comorbid group and depression group [3.40 (95% CI −1.81 to 8.60), *p* = 0.201]. These results show that treatment was associated with significant improvements overall (HADS total). These results appear to support the transdiagnostic effect associated with group MCT such that irrespective of reason for seeking treatment, there were significant decreases in levels of distress between pre- and post-treatment and follow-up.

#### Positive Metacognitive Beliefs

There was a significant main effect of time, with post-treatment and follow-up being associated with a respective 135.8 (95% CI 121.5–151.1) and 129.0 (95% CI 112.3–145.6) point reduction in positive metacognitive beliefs compared to baseline (*p* < 0.001), for the entire sample. There was a nonsignificant difference between groups on PMC at post-treatment, for depression compared to anxiety groups [−2.41 (95% CI −57.67 to 52.84), *p* = 0.932], for the comorbid compared to anxiety group [−16.04 (95% CI −13.70 to 45.78), *p* = 0.723], and comorbid compared to depression group [18.46 (95% CI −37.79 to 74.70), *p* = 0.520]. Likewise, at follow-up, there was a nonsignificant difference between the depression and anxiety groups [3.96 (95% CI −70.04 to 77.97), *p* = 0.916], the comorbid and anxiety groups [6.26 (95% CI −28.36 to 40.88), p = 0.723], and the comorbid compared to depression group [2.30 (95% CI −73.82 to 78.42), *p* = 0.953]. The results demonstrate that irrespective of reason for seeking treatment, MCT was associated with decreases in positive metacognitive beliefs. The results suggest changes in positive metacognitions were transdiagnostic and occur irrespective of reason for seeking treatment, MCT was associated with significant reductions in positive metacognitive beliefs between pre- and post-treatment and pre-treatment and follow-up.

#### Negative Metacognitive Beliefs

There was a significant main effect of time, with post-treatment and follow-up being associated with a 149.9 (95% CI 133.8–166.0) and 136.2 (95% CI 118.5–153.8) point reduction in NMC compared to baseline (*p* < 0.001) for the entire sample. There was a nonsignificant difference between groups on NMC at post-treatment, for depression compared to the anxiety group [41.70 (95% CI −31.21 to 114.60), *p* = 0.262], for the comorbid compared to anxiety [−0.34 (95% CI −33.05 to 32.36), *p* = 0.984] and compared to depression groups [−42.04 (95% CI 446.79–32.72), *p* = 0.270]. Likewise, at follow-up, there was a nonsignificant difference between the depression and anxiety groups [−7.26 (95% CI −53.50 to 38.97), *p* = 0.758], the comorbid and anxiety [−19.58 (95% CI −56.81 to 17.66), *p* = 0.303] and depression groups [−12.32 (95% CI −57.55 to 32.92), *p* = 0.594]. Overall, the results suggest improvement in negative metacognitions; however, this and the other results should be interpreted with caution due to the difference in number of individuals seeking treatment for anxiety and depression. Those with both anxiety and depression scored higher on NMC, 19 points (−4 to 53) although this was nonsignificant (*p* = 0.07). There were no significant group-by-time interactions in the analyses, suggesting that the nature of presenting problem did not modify outcomes.

## Sensitivity Analysis

All findings were robust under the sensitivity analysis where missing values at time 3 were mean imputed.

## Treatment Effect Sizes

The effect sizes (ES) associated with treatment were calculated based on Cohen’s *d* from pre- to post-treatment and pre-treatment to follow-up. Effect sizes were calculated based on subgroup (self-reported reason for seeking treatment; anxiety or comorbid) and for the total sample. Effect sizes were not calculated for the depression subgroup due to the small number of participants within this subgroup from pre-treatment to post-treatment (*n* = 12), and pre-treatment to follow-up (*n* = 8). All effect sizes are displayed in Table 3. Overall, the effect sizes are large, highlighting the potential efficacy of group MCT in a “transdiagnostic” sample. Between-subgroup effect sizes were calculated for the anxiety and comorbid subgroups at post-treatment for HADS total, positive metacognitive beliefs, and negative metacognitive beliefs. There was a small between-group effect size on the HADS total and positive metacognitive beliefs, Cohen’s *d* = 0.22 and 0.02, respectively, favoring the comorbid anxiety and depression subgroup. There was also a small to medium effect size difference, Cohen’s *d* = 0.37, on negative metacognitive beliefs favoring this subgroup. This highlights that there may be a slight advantage for individuals seeking treatment for anxiety and depression on outcomes but this may also be a function of greater initial severity in the comorbid cases.

**Table 3:** Treatment effect sizes based on treatment completers.

	Pre- to post-treatment	Pre-treatment to follow-up
**HADS total**		
Anxiety Comorbid Total sample	1.68 1.82 1.73	1.13 1.86 1.48
**PMC**		
Anxiety Comorbid Total sample	2.33 2.07 2.13	1.92 1.94 1.85
**NMC**		
Anxiety Comorbid Total sample	2.45 1.95 1.82	1.51 1.87 1.67

### Clinically Reliable Change

Reliable change was calculated for the total score of the Hospital Anxiety and Depression Scale for treatment completers. As the HADS has varying test-retest reliability scores and few have been calculated for the HADS total score, the average test-retest coefficient for the HADS total was calculated from Michopoulos et al. (2008) who reported a test-retest coefficient of 0.944 and from Spinhoven et al. (1997) who reported a test-retest coefficient of 0.91. Both test-retest coefficients were calculated over a 3-week interval. In order for patients to be classified as having made a reliable change, they had to have made at least a change of 6 points on the HADS total. A cutoff score of 15 was calculated using criterion “c” as outlined by Jacobson and Truax (1991) and used normative data from Crawford et al. (2001). Participants were classified as being improved if they made a reliable change but did not cross the cutoff, were classified as recovered if they made a reliable change and crossed the cutoff, were classified as no change if they did not make a reliable change, and as worsened if they reliably worsened. Table 4 outlines the number of participants that were classified at post-treatment and at 6-month follow-up. At post–treatment, 20.4% had made no change, 12.9% had improved, 66.7% had recovered, and none had worsened. At 6-month follow-up, 17.2% had made no change, 12.9% had improved, 65.6% had recovered, and 4.3% had worsened from pre-treatment to follow-up.

**Table 4:** Number and percentage of completers that reliably changed.

	Post treatment				6-month follow up			
	Anxiety n (%)	Depression n (%)	Comorbid n (%)	Total n (%)	Anxiety n (%)	Depression n (%)	Comorbid n (%)	Total n (%)
No change	12 (25.5%)	1 (12.5%)	6 (15.8%)	19 (20.4%)	11 (23.4%)	1 (12.5%)	4 (10.5%)	16 (17.2%)
Improved	2 (4.3%)	2 (25.0%)	8 (21.0%)	12 (12.9%)	3 (6.4%)	1 (12.5%)	8 (21.1%)	12 (12.9%)
Recovered	33 (70.2%)	5 (62.5%)	24 (63.2%)	62 (66.7%)	30 (63.8%)	6 (75%)	25 (65.8%)	61 (65.6%)
Worsened	0	0	0	0	3 (6. 4%)	0	1 (2.6%)	4 (4.3%)

## Discussion

Until now, most transdiagnostic interventions have not been derived from evidence-based generic models of psychological disorder that articulate common causal factors, but on pragmatic transdiagnostic manuals, which may have contributed to the small to moderate treatment effect sizes observed (Norton, 2008; Norton and Philipp, 2008; Newby et al., 2015). Therefore, we aimed to collate data from a mixed outpatient sample to assess the effects associated with transdiagnostic group MCT, which is based on a highly specified model. The treatment was associated with large effects that were consistent across patient subgroups and across measures. However, effect sizes should be treated with caution, as there was no comparison group. Irrespective of the participants’ reason for seeking treatment, the MCT intervention was associated with significant decreases in symptoms of anxiety and depression from pre- to post-treatment and these treatment gains were maintained at a group level over 6-month follow-up. Group MCT was associated with clinically significant changes with 80% of treatment completers having recovered or improved by post-treatment and 79% remaining recovered or improved at 6-month follow-up.

Group MCT has previously been evaluated in Generalized Anxiety disorder and Major Depressive disorder. van der Heiden et al. (2013) evaluated group MCT for individuals with GAD and found large (Cohen’s *d* = 2.01) pre-post treatment effect sizes on general anxiety. Similarly, group MCT has demonstrated large treatment effects for depression (Dammen et al., 2015); therefore, results from the current analysis are in line with previous studies evaluating group MCT.

In comparison to other trials of transdiagnostic treatment, the results from the current study offer promising support for the efficacy and potential superiority of group MCT in transdiagnostic groups. Effect sizes (ES) from previous transdiagnostic evaluations such as TD-CBT vary, ranging from small (Cohen’s *d* = 0.09, 0.20; Erickson et al., 2007; Norton and Barerra, 2012) to large (Cohen’s *d* = 0.93, 1.05, 1.15; Barlow et al., 2017 Laposa et al., 2017; Schmidt et al., 2012). While for mindfulness interventions, ES on symptoms of anxiety (hedge’s *g* = 0.08–0.56) and depression (hedge’s *g* = 0.22–0.59) (De Vibe et al., 2017) are low to moderate. In comparison, the current study demonstrated larger effect sizes at post-treatment ranging from Cohen’s *d* = 1.68 for individuals seeking treatment for anxiety to Cohen’s *d* = 1.82 for individuals with Norton both anxiety and depression. The results provide promising support for group MCT especially as the study was conducted within an unselected outpatient clinic. The results are also in line with previous studies of transdiagnostic evaluations of group MCT (Capobianco et al., 2018) that demonstrated large effects sizes at post-treatment (Cohen’s *d* = 1.38) and high recovery rates (71% of participants classified as improved at post-treatment).

The strengths of the current study include the use of a heterogenous group of patients and few exclusion criteria, meaning that the results have good generalizability to natural clinical settings. The large overall sample size provides a strong basis for generalizing to other groups of self-selected patients. The study however is not without its limitations. First, we did not use formal diagnoses as participants self-reported their reasons for seeking treatment and therefore we cannot determine whether the self-diagnoses actually represent *bona fide* disorders. However, the range in HADS scores, and scores on the PHQ-9 and GAD-7, show that patients were typically reporting levels of distress within the clinical range. A second limitation is the lack of a comparison or control group which means we cannot be sure that MCT was responsible for the improvement in symptoms and we cannot partial out the effects linked to time such as spontaneous remission. Spontaneous remission rates for anxiety are low (Bruce et al., 2005) while for depression spontaneous remission rates are high. Krøgsboll et al. (2009) found that 35% of improvement in depression could be attributed to spontaneous remission. However, given that the recovery rates for the study are higher than this at both post-treatment (67% recovered) and follow-up (66% recovered), the effects are much greater than would be expected form spontaneous improvements.

The preliminary findings from this study indicate that group transdiagnostic MCT in a sample of help-seeking patients with a mixture of psychological problems was associated with significant clinical gains that were not influenced by the nature of self-reported problems (or comorbidity). These results provide important pilot data for planning a more definitive randomized trial. If it can be substantiated that MCT is responsible for these effects, this treatment would constitute a cost-effective approach for treating mixed groups of patients suffering from a range of disorders.

## Ethics Statement

Ethical approval was not required for the study as per applicable institutional and national guidelines and regulations. Data were collected as part of routine clinical practice and evaluated as part of a service audit. However, in accordance with clinical guidelines, patients provided informed and written consent for use of patient data and ethical standards for reporting were adhered to. This is in line with the rules and regulations of the Danish National Ethics Committee.

## Author Contributions

PC, CJ, and SFN were responsible for data collection and delivery of the therapy. The study is part of the doctoral thesis completed by PC. LC was responsible for data entry and analysis and contributed to the write up of the manuscript. CH was responsible for data analysis and also contributed to the write up of the manuscript. AW was responsible for overall supervision of the study, analysis, and contributed to the write up of the manuscript.

### Conflict of Interest Statement

This evaluation was conducted for partial completion of the first author’s PhD, which was supervised by Professor AW.

The remaining authors declare that the research was conducted in the absence of any commercial or financial relationships that could be construed as a potential conflict of interest.
